# Conservative Management of Acute Anterior Cruciate Ligament Rupture in Recreational Athletes: A Pilot Case Series

**DOI:** 10.7759/cureus.103860

**Published:** 2026-02-18

**Authors:** María Balboa-Alonso, Jose-Manuel Gonzalo-Orden, David Corral-Fontecha, Susana Gomez-González, Luis Díaz-Gállego, Jorge Salvador-Marín, Oscar Balboa-Arregui

**Affiliations:** 1 Emergency Medicine, University Hospital San Juan de Alicante, Alicante, ESP; 2 Animal Medicine and Surgery, University of León, León, ESP; 3 Radiology, University Hospital of León, León, ESP; 4 Physical Anthropology, University of León, León, ESP; 5 Orthopedics and Traumatology, University Hospital of León, León, ESP; 6 Orthopedics and Traumatology, Hospital General Universitario Dr. Balmis de Alicante, Alicante, ESP; 7 Interventional Radiology, University Hospital of León, León, ESP

**Keywords:** anterior cruciate ligament, autologous conditioned serum, conservative treatment, mri, platelet-rich plasma, recreational athletes

## Abstract

Background and aim: Surgical anterior cruciate ligament reconstruction (ACLR) remains the standard treatment for acute anterior cruciate ligament (ACL) rupture in highly active individuals but entails surgical risks, prolonged recovery, and incomplete return to sport in some cases. Conservative approaches enhanced with biologics, such as platelet-rich plasma (PRP) or autologous conditioned serum (ACS), have gained interest as potential alternatives in selected patients. This study aimed to describe ligament healing, clinical stability, and short-term return to sport in recreational athletes with acute ACL rupture treated conservatively with intra-articular PRP or ACS plus structured rehabilitation.

Methods: This retrospective pilot case series included eight recreational athletes (four females, median age 32 years) with magnetic resonance imaging (MRI) and clinically confirmed acute ACL rupture (<4 weeks from trauma) who opted for non-operative care. All received four ultrasound-guided intra-articular injections of PRP (GPS III; Warsaw, IN: Zimmer Biomet) or ACS (Orthokine) over six weeks, followed by a standardized rehabilitation protocol. MRI evaluation at 21-24 weeks assessed ACL continuity (grade 0-3). Clinical assessment included the pivot-shift test graded according to the International Knee Documentation Committee (IKDC) grading system, instrumented Lachman testing, the Tegner Activity Scale (TAS), and the International Knee Documentation Committee (IKDC) score at 24 weeks.

Results: MRI showed restored ACL continuity in all patients (grade 0 in 7/8; grade 1 in 1/8). Post-treatment pivot-shift testing showed grade 0 in 7/8 patients and grade 1 in 1/8 patients, while the Lachman test was negative in all patients. All patients returned to recreational sports within six months post-injury. No re-ruptures or complications were observed during a minimum six-month follow-up after return to sport.

Conclusions: This study shows that intra-articular biologics (PRP or ACS) combined with structured rehabilitation were associated with MRI continuity, clinical stability, and safe return to sport at six months in recreational athletes with acute ACL rupture. This conservative treatment could be considered an alternative, and further investigation is warranted.

## Introduction

The anterior cruciate ligament (ACL) plays a critical role in stabilizing the knee joint by preventing anterior tibial translation and controlling rotational loads. ACL injuries are among the most common knee injuries in young and active individuals and are frequently associated with damage to menisci and articular cartilage, leading to long-term sequelae such as post-traumatic osteoarthritis (OA) [1-3].

While surgical ACL reconstruction (ACLR) remains the gold standard for restoring stability and function in highly active populations, not all patients are candidates for or willing to undergo surgery. Additionally, not all individuals return to their pre-injury level of sport, and surgery carries known complications, prolonged rehabilitation, and high socioeconomic burden [4-7]. In recent years, conservative management strategies, including bracing and structured rehabilitation, have gained renewed interest. Some prospective studies suggest that a subset of patients, especially those with low-demand functional profiles, may achieve sufficient knee stability and functional recovery without surgery [8-10].

In parallel, the use of biologic agents such as platelet-rich plasma (PRP) and autologous conditioned serum (ACS) has expanded across musculoskeletal medicine, including ligament injuries [10-12]. These treatments aim to harness anti-inflammatory cytokines and growth factors to promote endogenous healing and modulate the intra-articular environment. However, most studies on biologics in ACL injury focus on surgical augmentation or graft maturation rather than conservative, standalone therapy [13-17].

MRI-based studies suggest that ACL continuity may be restored spontaneously in a small proportion of non-operatively treated cases [18]. Emerging data indicate that intra-articular biological treatments might accelerate this reparative process or improve clinical outcomes in selected populations [18-25]. Still, clinical evidence remains sparse, heterogeneous, and often limited by small sample sizes, lack of controls, and product variability.

This study presents a retrospective pilot case series of recreational athletes with acute ACL rupture who elected non-operative management with PRP or ACS injections and a standardized rehabilitation protocol. Our primary aim was to describe the MRI appearance of the ACL at six months. Secondary outcomes included clinical knee stability, return to sport, and safety. This preliminary investigation aimed to generate hypotheses for future controlled studies on conservative biologic strategies in ACL injury.

## Materials and methods

Study design and ethics

This was a retrospective observational case series conducted at two tertiary centers between March 2023 and August 2024. All patients provided written informed consent for treatment and for the use of anonymized data and imaging for research and publication. This study involved secondary analysis of fully anonymized clinical data collected during routine care, with no additional interventions. In accordance with institutional policy, such studies are exempt from formal ethics committee approval. The study adhered to the principles of the Declaration of Helsinki.

Patient selection

Patients were eligible for inclusion in this study if they met the criteria in Table 1. Patients chose conservative management after discussion with an orthopedic specialist. The rupture pattern (complete vs. partial) and location (femoral, tibial, mid-substance) were characterized using the Anterior Cruciate Ligament OsteoArthritis (ACLOAS) criteria [26,27].

**Table 1:** Inclusion and exclusion criteria. ACL: anterior cruciate ligament; MRI: magnetic resonance imaging

Inclusion criteria	Exclusion criteria
Age 18-55 years	Prior ACL surgery on the affected knee
Recreational athletes (Tegner score 6-7)	Multiligament or meniscus injury requiring surgical repair
Acute ACL rupture diagnosed within 4 weeks of trauma	Fracture
MRI confirmation of rupture, plus positive clinical instability tests (pivot-shift and instrumented Lachman)	Infection
-	Inability to complete the structured rehabilitation protocol

Injection protocol

All patients received a series of four ultrasound-guided intra-articular injections, administered at two-week intervals, starting within weeks two to three post-injury. Each injection consisted of 5 mL of either autologous conditioned serum (ACS) prepared using Orthokine (Orthogen, Germany) or platelet-rich plasma (PRP) prepared using GPS III (Warsaw, IN: Zimmer Biomet).

Both products were processed strictly following the manufacturer's instructions. No laboratory analysis of composition (e.g., platelet/leukocyte count, growth factor/cytokine concentration) was performed. The choice between PRP and ACS was not randomized. In clinical practice, patients selected their treatment after a shared decision-making discussion with the treating physician, based on availability and logistical considerations. No predefined clinical or imaging criteria were used to allocate one product over the other, reflecting pragmatic real-world application but limiting comparability. This pilot study does not attempt to compare the results of PRP and ACS.

Rehabilitation protocol

A standardized rehabilitation program was initiated for all patients. Key phases were included in Table 2. Patients were evaluated before returning to sport by a multidisciplinary team that included orthopedic surgery, sports medicine, and radiology.

**Table 2:** Standardized weekly rehabilitation program. MRI: magnetic resonance imaging

Weeks	Rehabilitation program
Weeks 0-2	Protected range of motion, limited weight-bearing with a brace
Week 4	Ergometric cycling, walking, and proprioceptive exercises
Week 8	Initiation of jogging and progressive strength work
Weeks 16-18	Plyometric exercises, sports-specific drills
Week 24	Return to recreational sport, conditional on clinical and MRI criteria

Clinical assessment

Clinical instability was assessed at baseline and after treatment according to the following parameters: (1) Pivot displacement test - scored from 0 to 3 according to the International Knee Documentation Committee (IKDC) grading system. (2) Instrumented Lachman test - assessed using the Knee Laxity Tester (KLT) arthrometer; the result was considered negative if the side-to-side difference was <3 mm, and the absolute anterior tibial translation was <10 mm. (3) The Tegner Activity Scale (TAS) was recorded before and after the injury to assess return to recreational activity [28,29]. Return to sport was assessed based on patient self-report during follow-up visits, supported by clinical examination, functional capacity assessment, and the IKDC score at 24 weeks.

MRI acquisition and evaluation

MRI scans were obtained at baseline and follow-up (21-24 weeks). All patients were imaged on 1.5 T systems with proton-density (PD) and PD fat-saturated (PD-FS) sequences in sagittal, coronal, and axial planes. Ligament continuity was graded using a four-point system (Table 3).

**Table 3:** MRI ligament continuity. ACL: anterior cruciate ligament.

Grade	Ligament continuity in MRI
Grade 0	Continuous low-signal ACL with normal thickness
Grade 1	Continuous with increased signal
Grade 2	Continuous but thin/lax
Grade 3	Discontinuous/absent

MRI scans were reviewed by a single musculoskeletal radiologist with over 10 years of experience, who was blinded to clinical outcomes and baseline imaging during follow-up at the time of interpretation. A structured grading system was applied to classify ACL appearance (grades 0-3) based on continuity, signal intensity, and morphology [26,27,30]. Associated findings, such as bone bruising, meniscal, or chondral lesions, were also recorded.

Statistical analysis

Given the small sample size and pilot nature of the study, only descriptive statistics were used. Continuous variables are reported as median and interquartile range (IQR), or mean with standard deviation (SD) if symmetric. Categorical variables are presented as counts and percentages. No hypothesis testing was performed.

## Results

Baseline characteristics

Eight recreational athletes were included (four female and four male), aged 21-50 years (median age: 32 years, IQR: 28-41). All had MRI and clinically confirmed ACL ruptures and chose non-surgical management with either PRP (n=4) or ACS (n=4), followed by a standardized rehabilitation protocol. Most injuries occurred during football or padel. All presented with a positive Lachman test and pivot-shift grade 2 at baseline. MRI grading showed complete rupture in all cases, most frequently at the femoral insertion (Table 4).

**Table 4:** Patient characteristics and diagnosis. ACL: anterior cruciate ligament; MRI: magnetic resonance imaging; Lachman (+): positive instrumented laxity; TAS: Tegner Activity Scale

Patient number	Age (years)	Sex	Sports	TAS pre/injury	Pivot-shift grade	Lachman test	MRI (ACL injury grade)	MRI grade/location
1	50	F	Ski	7/2	2	+	2	III/femoral
2	32	F	Padel	6/2	2	+	2	III/femoral
3	31	M	Padel	6/1	2	+	2	III/intra-ligamentary
4	21	F	Golf	6/2	3	+	3	III/tibial
5	41	M	Football	6/2	2	+	3	II-III/intra-ligamentary
6	38	M	Football	6/1	3	+	3	III/femoral
7	44	F	Football	6/2	2	+	2	III/femoral
8	26	M	Handball	6/1	2	+	2	III/femoral

Post-treatment MRI and clinical outcomes

**Figure 1:**
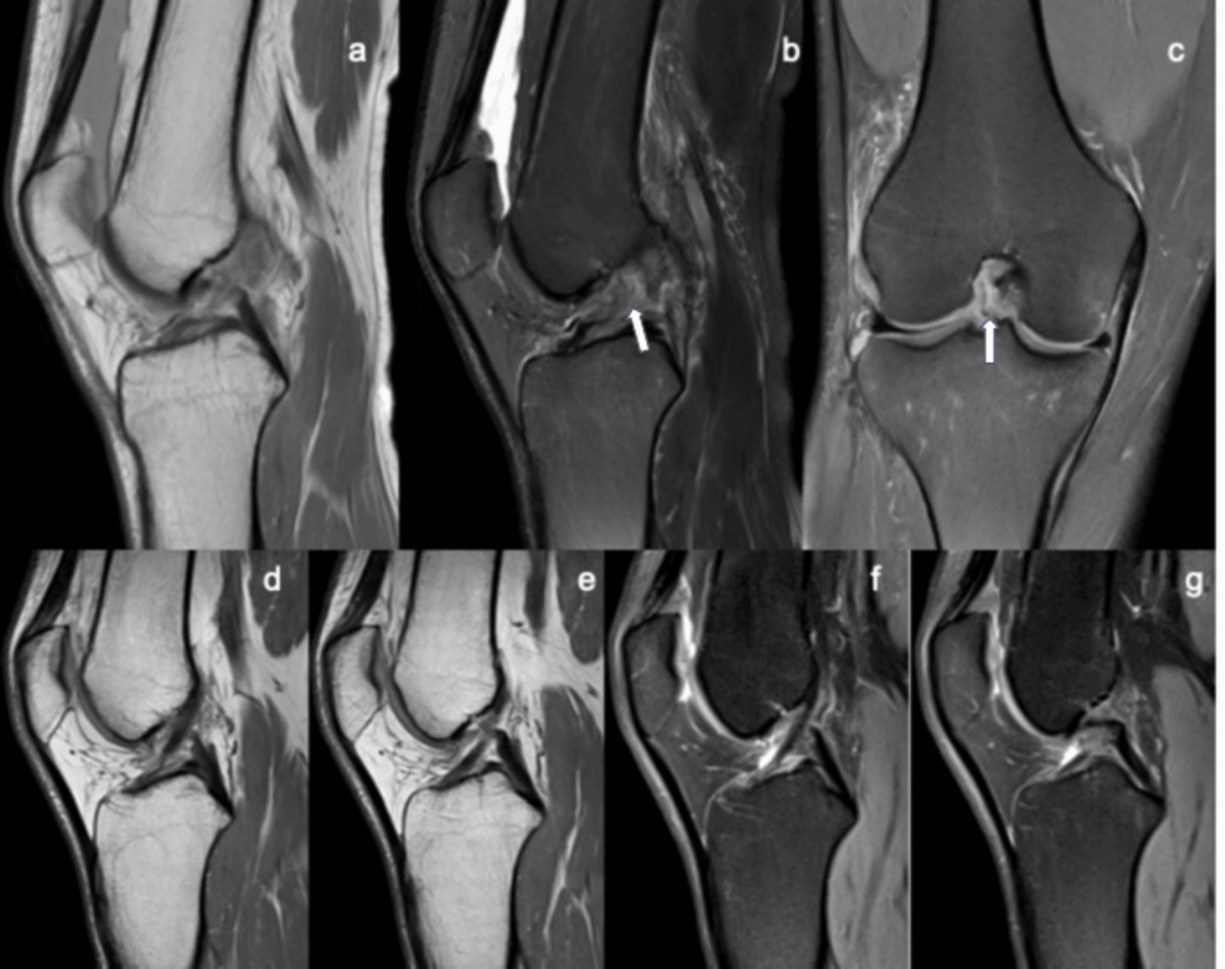
Magnetic resonance imaging 10 days after ACL injury and after 22 weeks of biological conservative treatment. Case 2: magnetic resonance imaging baseline proton density sagittal (a), proton-density fat-saturated sagittal (b), and coronal (c) at 10 days shows grade III ACL rupture (white arrows). Follow-up at 22 weeks post-PRP treatment - anterior cruciate ligament continuity and low signal are observed (repair grade 0) (d-g). PRP: platelet-rich plasma; ACL: anterior cruciate ligament

**Figure 2:**
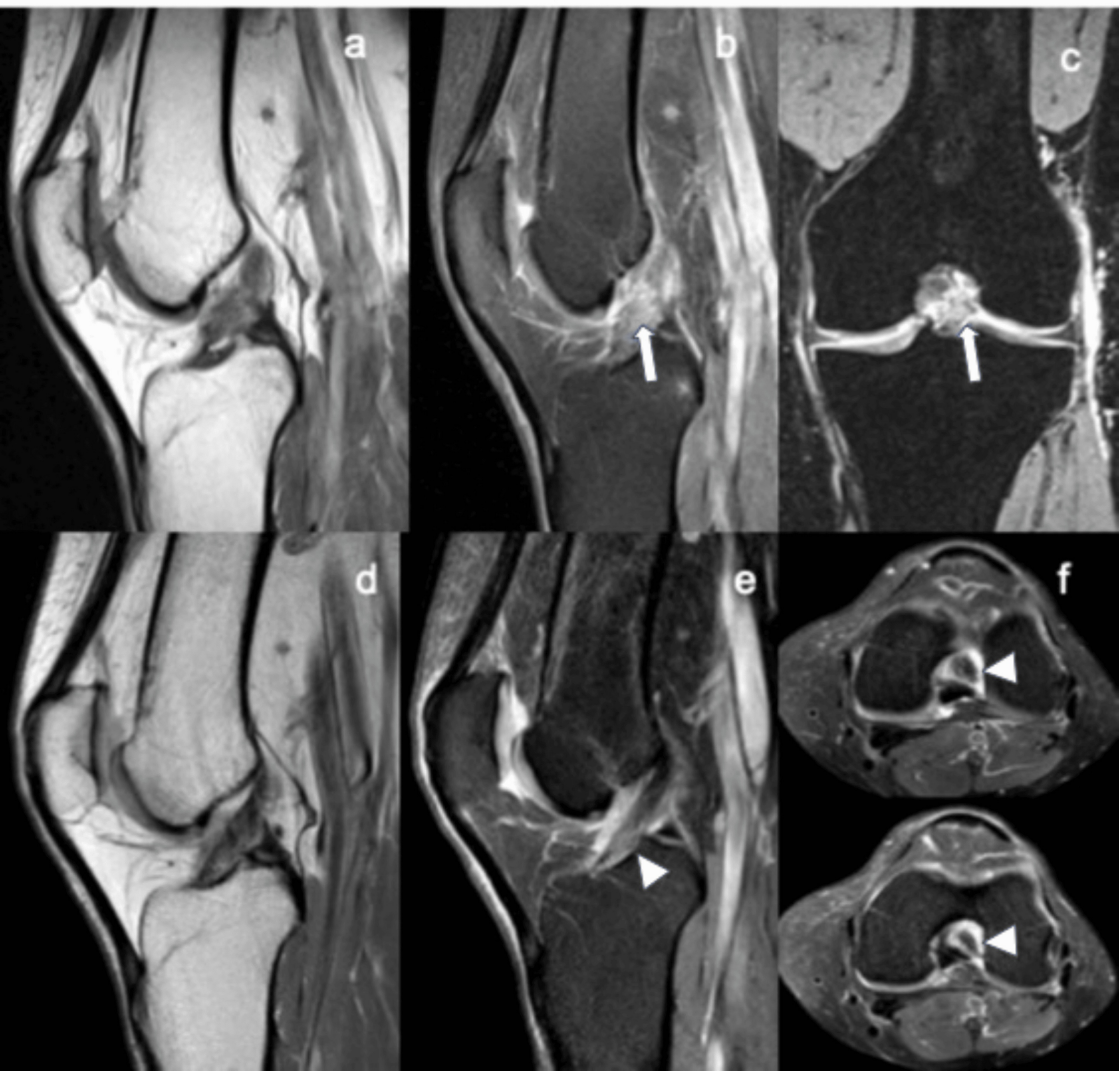
Magnetic resonance imaging eight days after knee trauma and after 24 weeks of conservative biological treatment. Case 5: magnetic resonance imaging baseline at eight days after trauma shows grade II-III anterior cruciate ligament rupture (white arrows) and grade I medial collateral ligament injury (a-c). Follow-up at 24 weeks post-ACS shows continuous anterior cruciate ligament (arrowhead) with high signal in the lower third (repair grade 1) (d-f). ACS: autologous conditioned serum

**Figure 3:**
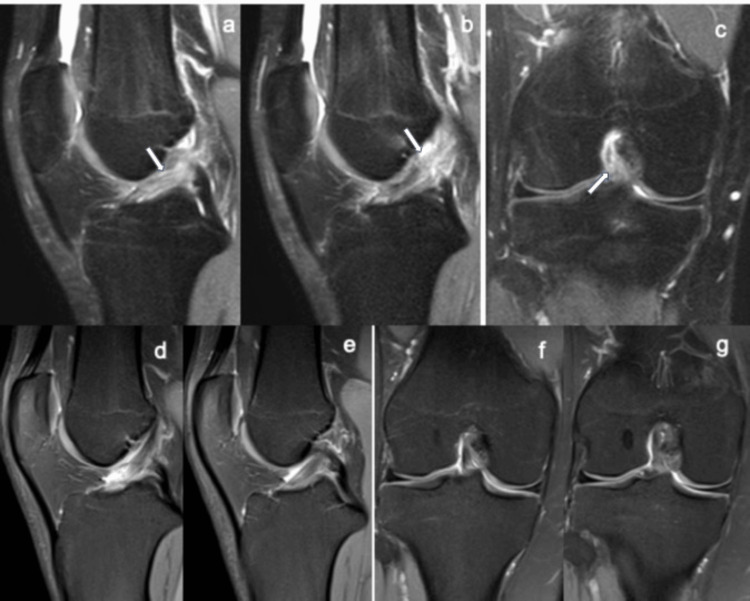
Magnetic resonance imaging 12 days after ACL injury and after 21 weeks of conservative biological treatment. Case 8: baseline magnetic resonance imaging at 12 days post-injury shows a grade III anterior cruciate ligament rupture (white arrows in a-c). Follow-up magnetic resonance imaging at 21 weeks post-PRP treatment shows restored anterior cruciate ligament continuity (repair grade 0) on sagittal (d, e) and coronal proton density fat-saturated (f, g) sequences. PRP: platelet-rich plasma

All patients underwent follow-up MRI between weeks 21 and 24. Ligament continuity was restored in all cases: seven were classified as grade 0 (normal low-signal continuous ligament) and one as grade 1 (high-signal but continuous). Post-treatment clinical evaluation showed negative Lachman in all patients (anterior tibial translation {ATT} <3 mm) and pivot-shift grade 0 in 7/8 patients. Table 5 summarizes the post-treatment clinical outcomes, MRI repair grades, and follow-up duration for each patient. Only one patient had residual pivot grade 1. Representative examples of MRI evolution are shown in Figures 1a-1g, 2a-2f, 3a-3g.

**Table 5:** Results after intra-articular PRP treatment. ACS: autologous conditioned serum; IKDC: International Knee Documentation Committee; MCL: medial collateral ligament; PRP: platelet-rich plasma; Repair-grade MRI: MRI at 21-24 weeks; TAS: Tegner Activity Scale

Patient number	Product	Pivot (post)	Lachman test	Repair-grade MRI	Other MRI findings	TAS/IKDC 6 months	Follow-up (months)
1	PRP (GPS III)	0	-	0	Cartilage thinning	7/96	12
2	PRP (GPS III)	0	-	0	-	6/100	12
3	ACS (Orthokine)	0	-	0	-	6/92	11
4	ACS (Orthokine)	1	-	0	Grade 1/MCL injury	6/92	10
5	ACS (Orthokine)	0	-	1	-	5/88	9
6	PRP (GPS III)	0	-	0	-	6/95	8
7	ACS (Orthokine)	0	-	0	Cartilage thinning	6/100	8
8	PRP (GPS III)	0	-	0	-	6/96	6

Return to sport and safety outcomes

All patients resumed their pre-injury recreational sport by week 24. Tegner Activity Scale returned to baseline in 7/8 and decreased by one point in one case. The IKDC score was >85 in 8/8 patients. No complications or re-ruptures were recorded during the follow-up period (six to 12 months post-return).

## Discussion

This pilot series suggests that intra-articular administration of PRP or ACS, combined with a structured rehabilitation protocol, may support ligament continuity and functional stability in recreational athletes with acute ACL rupture who opt for conservative treatment. MRI evidence of ligament continuity, normalization of pivot-shift and Lachman tests, and return to sport were observed in all patients within six months, without complications or re-ruptures during follow-up.

Spontaneous recovery of ACL continuity has been described in selected cases managed non-operatively with bracing and rehabilitation, particularly for proximal or partial tears [4,6,7,9,30]. Secondary analyses from the Knee Anterior Cruciate Ligament Non-surgical vs Surgical Treatment Trial (KANON Trial), a landmark randomized controlled trial comparing early ACL reconstruction with rehabilitation and optional delayed surgery, reported that ACL MRI continuity may be associated with better patient-reported outcomes, even without reconstruction [30]. These findings support the hypothesis that the native ACL has intrinsic healing capacity in select patients.

Biologic injections have also been explored in the post-operative setting to accelerate graft maturation or reduce inflammation after ACL reconstruction, with mixed results [16,17,22,25]. In contrast, studies on biologics as part of primary conservative management are scarce, and most available data come from small case series or case reports [19-21,24,25]. Our findings align with this limited body of evidence and add new data on a specific group, recreational athletes with acute ruptures, who often fall in a gray zone between high-performance demands and conservative eligibility. Notably, the rate of return to recreational sport in our cohort (100% by week 24) and the absence of re-ruptures during short-term follow-up are comparable to those reported in some surgical series in similar populations.

Overall, our results support the notion that in highly selected patients, structured rehabilitation combined with biologic support may represent a viable alternative to early ACL reconstruction. Further studies are needed to identify predictors of successful non-operative management and to clarify the role of intra-articular biologics within this paradigm.

These results suggest that, in carefully selected recreational athletes, particularly those with moderate activity levels and proximal rupture patterns, conservative management with intra-articular biologic support may be considered as part of the shared decision-making process. Patients should be informed of the limited evidence, potential risks, and need for structured rehabilitation and close monitoring.

Although PRP and ACS are increasingly used in orthopedics, the lack of standardization in preparation, dosage, injection protocols, and outcome reporting limits their clinical adoption. Our experience highlights the feasibility and tolerability of ultrasound-guided intra-articular administration in an outpatient setting.

Limitations and future directions

This study has several important limitations. First, it is a retrospective level IV case series with a small sample size (n=8) and no control group. As such, no causal inference can be made regarding the efficacy of PRP or ACS. The observed improvements may reflect the natural course toward ligament continuity with structured rehabilitation, as reported in previous literature. The potential placebo effect of intra-articular injection and the psychological impact of active treatment cannot be excluded.

Second, treatment allocation was not randomized. All patients opted for conservative management after being informed of surgical and non-surgical options. This introduces self-selection bias, as patients motivated to avoid surgery may also adhere better to rehabilitation, potentially skewing outcomes.

Third, two different commercial systems were used (PRP: GPS III; ACS: Orthokine), without product characterization (e.g., platelet counts, leukocyte content, cytokine levels). No predefined rationale was used for product selection; it was based on patient preference and logistical availability. As such, reproducibility is limited, and biological exposure cannot be precisely quantified.

Fourth, MRI grading of ligament continuity was performed by a single musculoskeletal radiologist, with no formal interobserver reliability assessment. Clinical testing (pivot-shift, Lachman, and IKDC score) was performed by treating clinicians without blinding. Additionally, validated patient-reported outcome measures (e.g., Knee Injury and Osteoarthritis Outcomes Score {KOOS}, Lysholm) were not collected, limiting functional interpretation. Finally, follow-up was limited to six to 12 months after return to sport. Long-term outcomes, such as reinjury, meniscal degeneration, or osteoarthritis progression, could not be evaluated within this timeframe.

Prospective studies are needed to compare conservative biological treatment with standard ACL reconstruction or structured rehabilitation alone without biological treatment. Standardized protocols for biologic preparation and administration, validated outcome measures, and longer follow-up should be adopted.

Multicenter registries or randomized trials may help quantify effect size, define ideal candidates, and evaluate cost-effectiveness. The integration of MRI-based tissue biomarkers, gait analysis, and patient-reported outcomes would enhance clinical relevance.

## Conclusions

In this hypothesis-generating pilot series of recreational athletes with acute ACL rupture, conservative management with intra-articular PRP or ACS, combined with structured rehabilitation, was associated with MRI evidence of ligament continuity, clinical stability, and return to recreational sport within six months. These preliminary results are promising and contribute to the growing interest in biologic-enhanced conservative approaches. Further prospective studies with standardized protocols and control groups will be valuable for confirming the durability of these outcomes and better defining the roles of PRP and ACS in non-surgical ACL treatment.
